# A Case of Bilateral Acro-Osteolysis Following Osteomyelitis in a Patient With Type 2 Diabetes Mellitus and a Literature Review on Acro-Osteolysis

**DOI:** 10.7759/cureus.83071

**Published:** 2025-04-27

**Authors:** Mohamed F Ahmed, Sohaib Eladl, Ee Tienne Ong, Portia N Mzezewa

**Affiliations:** 1 Department of Diabetes and Endocrinology, Blackpool Teaching Hospitals NHS Foundation Trust, Blackpool, GBR; 2 Department of Accident and Emergency, Blackpool Teaching Hospitals NHS Foundation Trust, Blackpool, GBR; 3 Department of Internal Medicine, Blackpool Teaching Hospitals NHS Foundation Trust, Blackpool, GBR; 4 Department of Respiratory Medicine, Blackpool Teaching Hospitals NHS Foundation Trust, Blackpool, GBR

**Keywords:** acro-osteolysis, endocrinology and diabetes, orthopaedics & traumatology, osteomyelitis, type 2 diabetes

## Abstract

Acro-osteolysis is a rare condition characterised by progressive resorption of the distal phalanges, often associated with systemic diseases, neuropathy, or vascular dysfunction. While commonly linked to autoimmune and rheumatic conditions, its occurrence in diabetic patients following osteomyelitis is infrequent.

We present a case of bilateral acro-osteolysis in a male in his mid-70s with type 2 diabetes mellitus and a history of chronic osteomyelitis. Despite appropriate antimicrobial management, the patient exhibited ongoing bone resorption in both feet. A comprehensive autoimmune and vascular workup ruled out alternative causes, supporting the diagnosis of acro-osteolysis secondary to chronic infection.

This case highlights the diagnostic challenges and potential mechanisms of acro-osteolysis in diabetic patients. Early recognition and a multidisciplinary approach are crucial to preventing functional disability.

## Introduction

Acro-osteolysis is a rare condition characterised by progressive resorption of the distal phalanges, typically linked to systemic diseases, infections, neuropathy, or vascular dysfunction. It is infrequently reported in diabetic patients, where chronic osteomyelitis can contribute to bone destruction despite appropriate medical management. We present a rare case of bilateral acro-osteolysis in a patient with long-standing type 2 diabetes mellitus (T2DM), where osteolysis persisted despite the resolution of active infection. Comprehensive investigations ruled out alternative autoimmune and dermatological causes [1].

## Case presentation

A male patient in his mid-70s with a medical background of T2DM, chronic obstructive pulmonary disease (COPD), pancreatitis, and prostate cancer presented to the emergency department (ED) in June 2022, with bilateral foot ulcers that were painful and non-healing, with progressive swelling and deformity. The duration of the ulcers was unknown to the patient. However, pain and redness flared up the day before admission, especially around the right big toe. Infection markers were raised on admission (C-reactive protein: 178; white cell count: 12.4).

**Figure 1:**
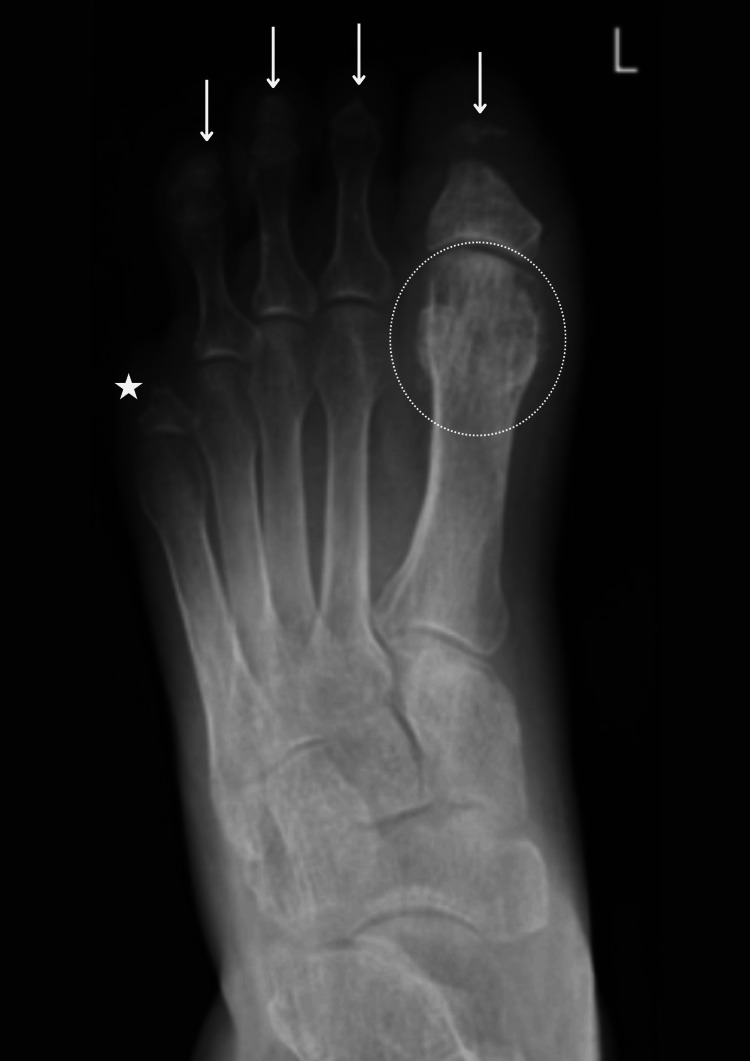
Left foot X-ray (June 2022). Active osteomyelitis of the first proximal phalanx and remaining digits. As demonstrated, there is notable soft tissue swelling of the left hallux, with an absent distal phalanx and deformity of the proximal phalanx head/neck. In addition, the left second distal phalanx is absent, with tapering of the middle phalanx, and there is deformity of the third and fourth distal phalanges. These appearances are considered secondary to active osteomyelitis, given the surrounding soft tissue swelling. Amputation of the fifth digit at the level of the proximal shaft is noted; the amputation site appears stable.

**Figure 2:**
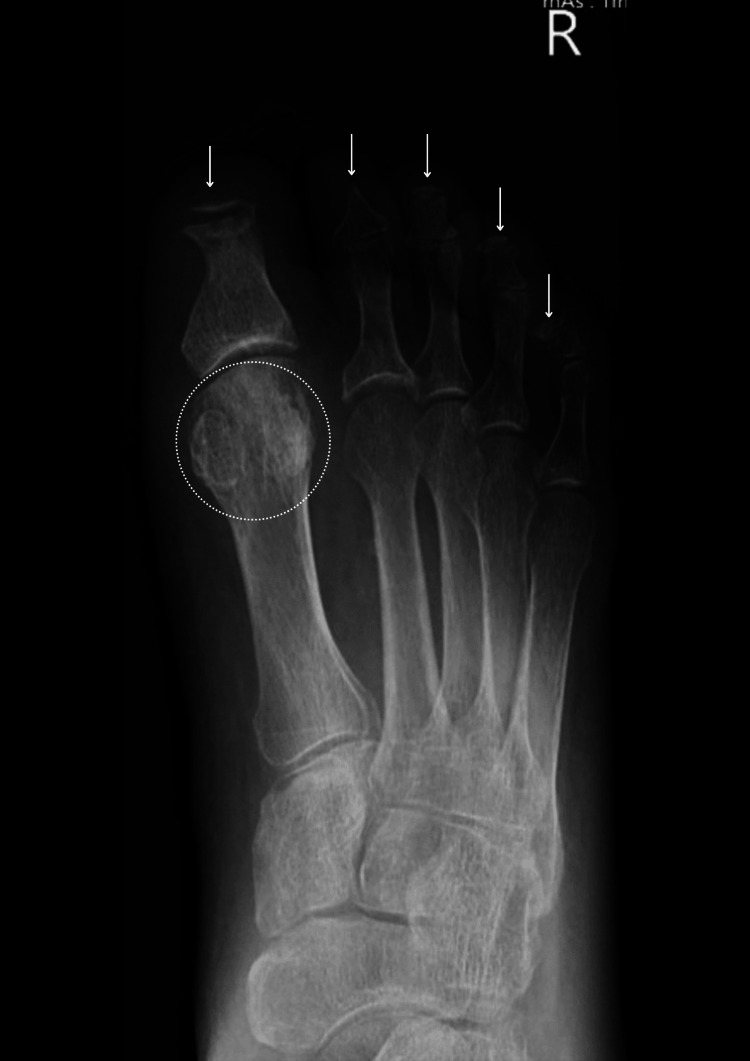
Right foot X-ray (June 2022). Active osteomyelitis of the first proximal phalanx and remaining digits. Notable soft tissue swelling of the right hallux with absent distal phalanx and deformity of the proximal phalanx head/neck. In addition, the right 2nd distal phalanx is also absent, with tapering of the middle phalanx, and there is also deformity of the right 3rd, 4th, and 5th distal phalanges. Overall appearances are considered secondary to active osteomyelitis, given the surrounding soft tissue swelling.

Radiographic evaluation was performed, and this showed ongoing active osteomyelitis of the first proximal phalanx and remaining digits (Figures 1, 2).

Follow-up imaging

**Figure 3:**
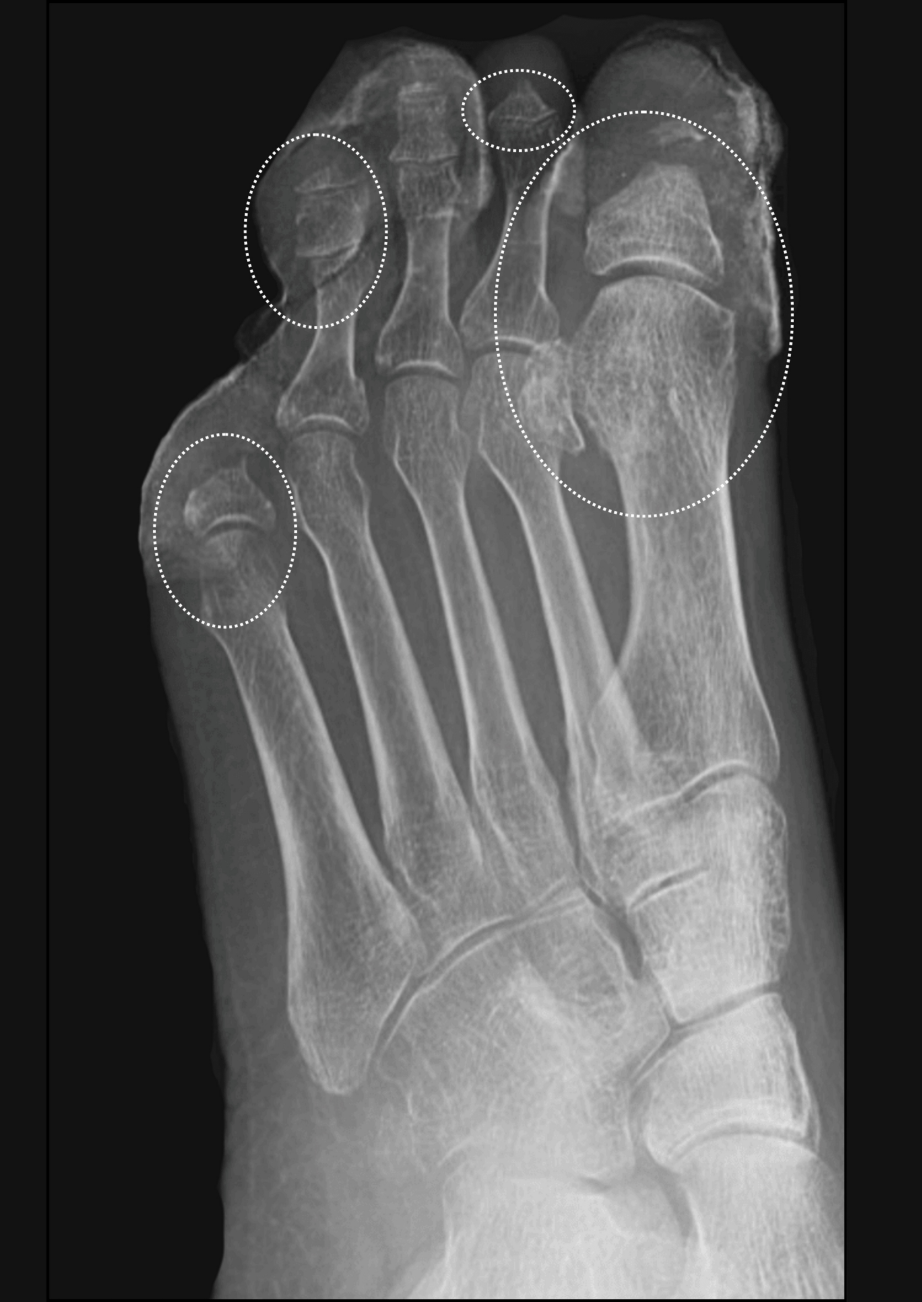
Left foot X-ray showing potential osteomyelitis in the annotated sites. Compared with a previous scan done in June 2022, there is ongoing ostial lysis of the great toe distal and proximal phalanx. The tips of the distal phalanx are phalanges of the fourth and fifth toes, the middle phalanx of the second toe, and the proximal phalanx of the fifth toe. In addition, there is new periarticular osteopenia in the metatarsal distally of the great toe and the fifth toe, as well as the second toe, which raises the possibility of osteomyelitis at these sites.

**Figure 4:**
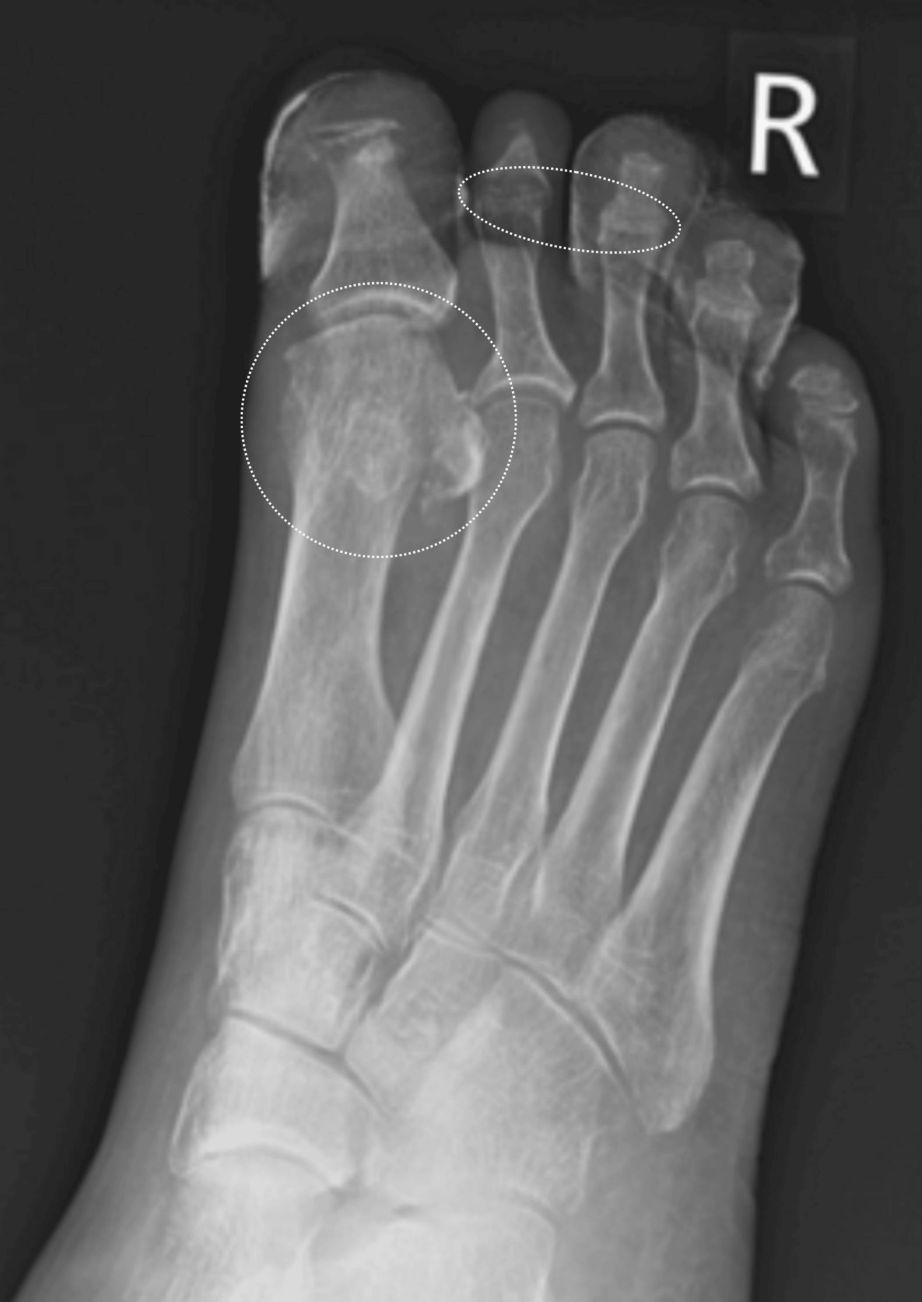
Right foot X-ray done in July 2022. Compared with June 2022, there is ongoing osteolysis of the distal phalanges of the toes and middle phalanges of toes 2 and 3 and the proximal phalanx of the first toe.

A repeat X-ray in July 2022 showed progressive osteolysis. When compared to the findings in June 2022, it is revealed that on the left foot, there is ongoing progressive osteolysis and new periarticular osteopenia (Figure 3), while in the right foot, there is new periarticular osteopenia (Figure 4). These suggest ongoing osteomyelitis.

Vascular assessment

**Figure 5:**
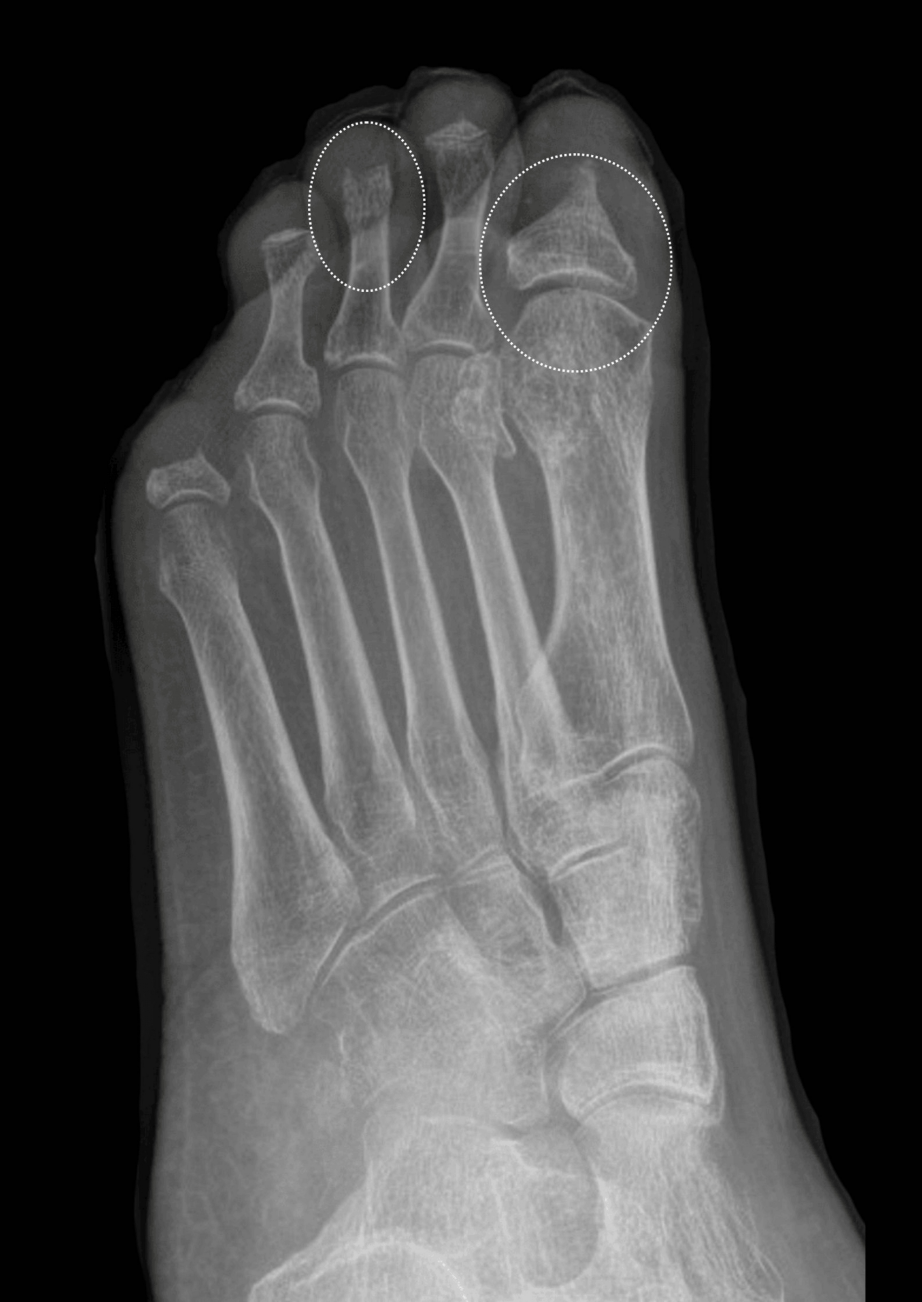
X-ray of the left foot. Further amputation of the left toes noted. There is some cortical irregularity at the 1st and 3rd amputation sites that may suggest underlying low-grade osteomyelitis.

**Figure 6:**
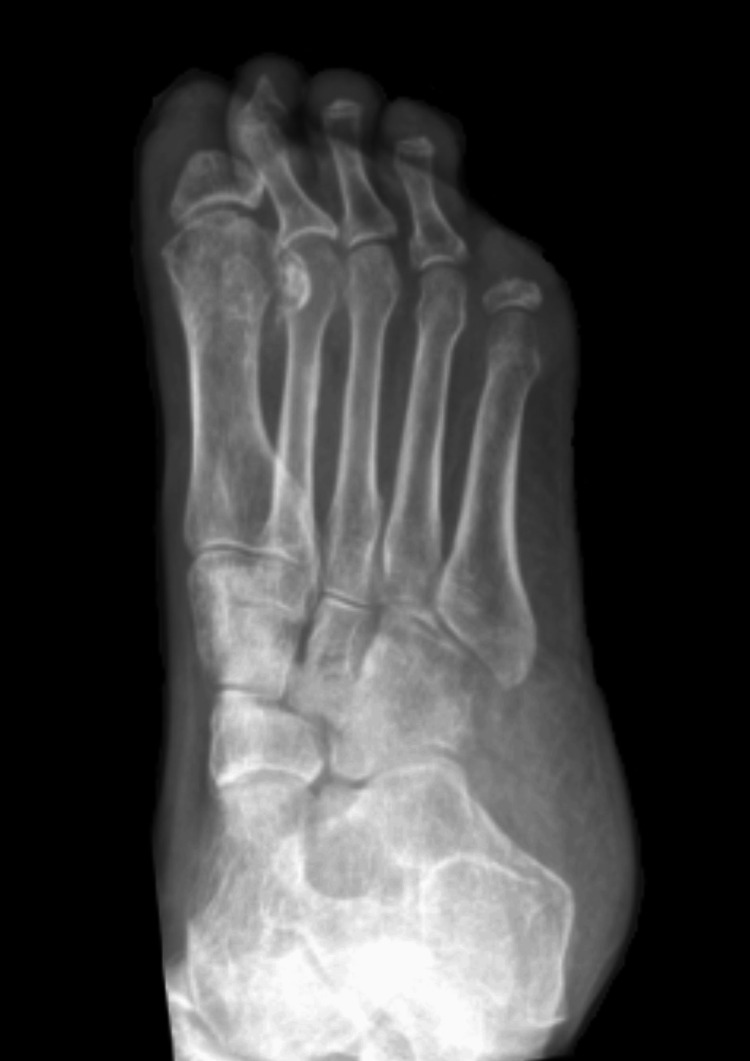
Comparison X-ray of the right foot. Further amputation of the right toes noted, with stable appearances of all the amputation sites. No plain film evidence of osteomyelitis was noted.

A duplex ultrasound arterial-brachial pressure index (ABPI) study (July 2022) revealed no significant arterial disease in both lower limbs. A follow-up X-ray done in February 2023 showed progressive improvement in features of osteomyelitis (Figures 5, 6).

A magnetic resonance angiography (MRA) of the lower limbs (August 2023) showed moderate disease in both superficial femoral arteries, but was otherwise unremarkable.

Surgical intervention and further progression

**Figure 7:**
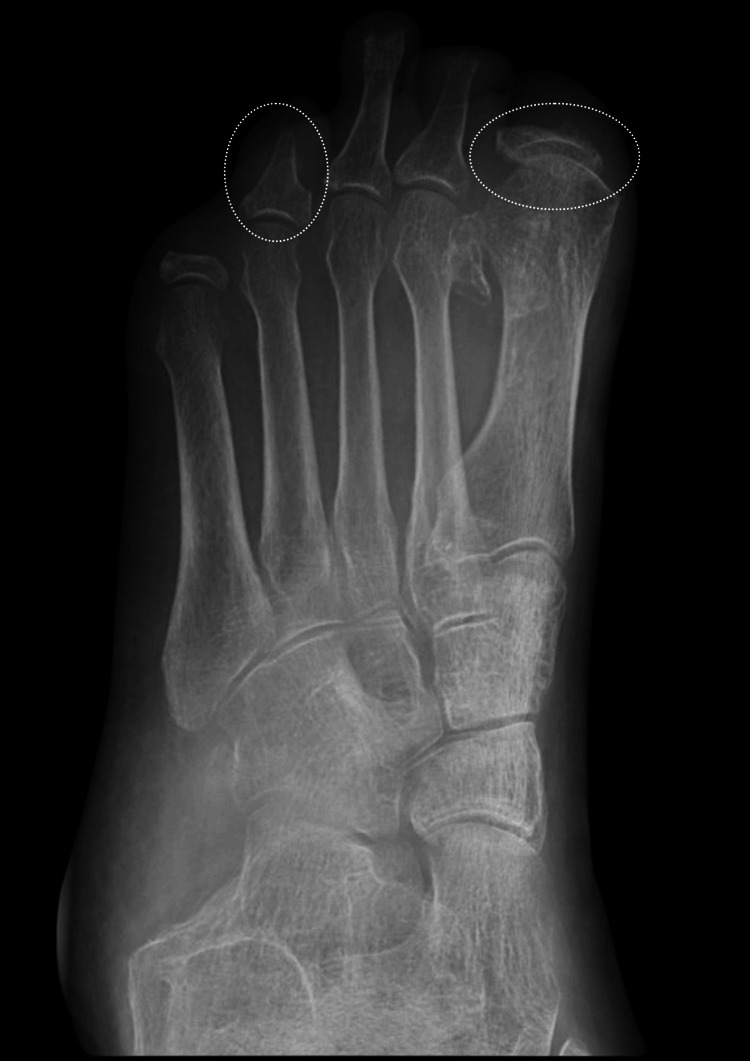
Left foot X-ray done in September 2023. There is more resorption of the bone of the proximal phalanx big toe compared to the last radiograph. Small erosion in the second and third toes is unchanged. There is resorption of the bone in the fourth toe since the last radiograph, with a stable appearance of the fifth toe.

**Figure 8:**
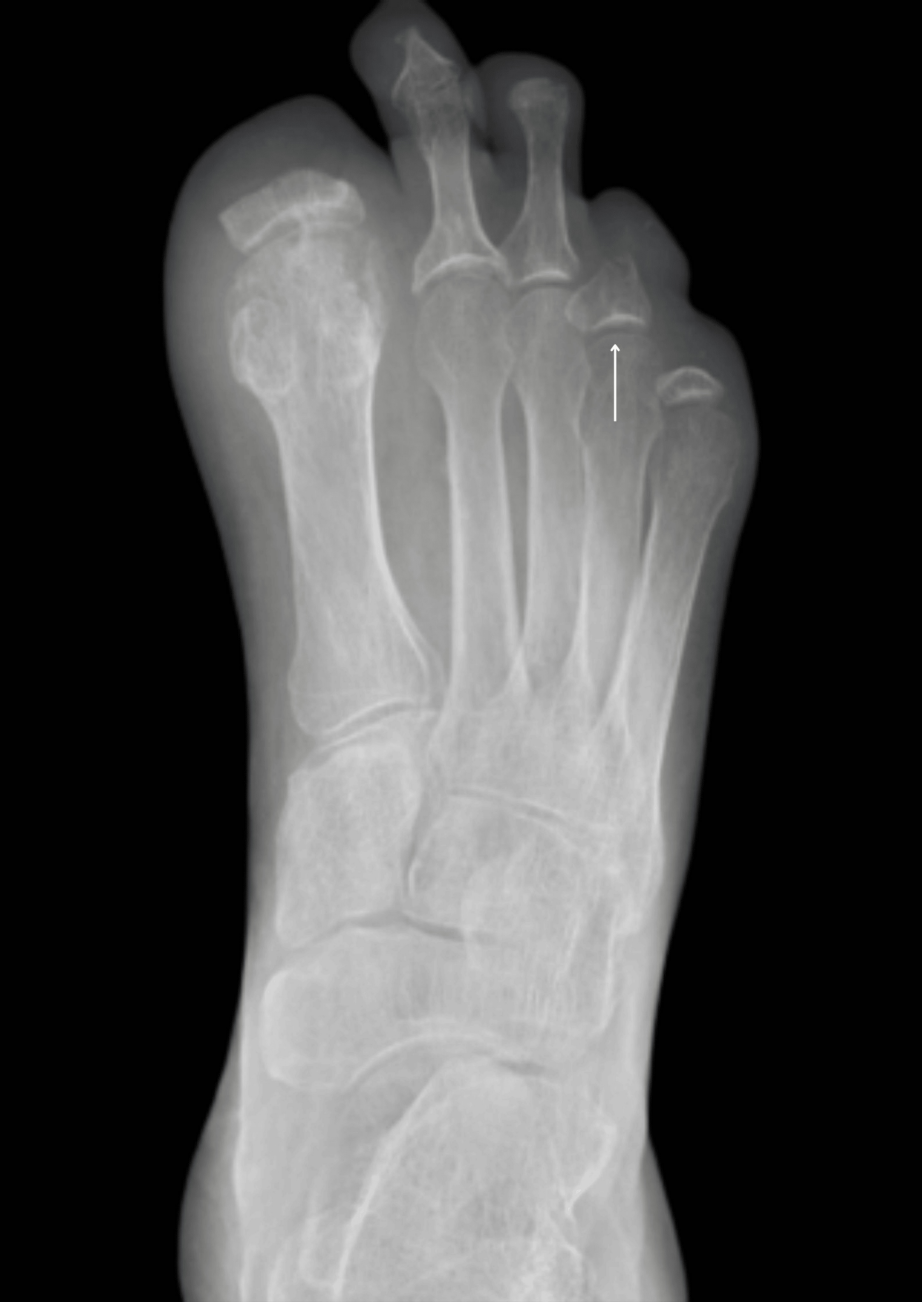
Right foot X-ray done in September 2023. Unchanged appearance of the amputated big toe. The second and third toes are unchanged in appearance. There appears to be resorption of the proximal phalanx of the fourth toe, which suggests some ongoing osteomyelitis. The fifth toe's appearance is unchanged.

In September 2023, the patient underwent debridement of the right big toe due to osteomyelitis. Subsequent imaging postoperatively showed ongoing bone resorption (Figures 7, 8).

Investigation for dermatological and autoimmune conditions

Given the unusual progression of osteolysis, a thorough workup was conducted to exclude autoimmune and dermatological conditions, including scleroderma. The following tests were negative: *Aspergillus* IgE (quantitation), human leukocyte antigen (HLA)-B27, IgA antibody to endomysium, liver/kidney microsomal antibodies (LKM), rheumatoid factor (latex screen), reticulin (R1) antibodies, IgA antibodies to tissue transglutaminase, and a connective tissue disease screen. These results effectively ruled out conditions commonly associated with osteolysis.

Diagnosis

Based on the clinical course, imaging, and exclusion of secondary causes, the patient was diagnosed with acro-osteolysis secondary to chronic osteomyelitis.

## Discussion

Acro-osteolysis is a rare complication in diabetic patients and may occur despite well-controlled diabetes and resolved osteomyelitis on imaging. The progressive bone resorption observed in this patient highlights the potential for ongoing bone loss even in the absence of active infection. The exact mechanisms remain unclear but may involve a combination of vascular insufficiency, autonomic neuropathy, and an exaggerated inflammatory response.

Early recognition and aggressive management, including vascular optimisation, infection control, and possible reconstructive interventions, are crucial to preventing functional disability in such patients.

Literature review

Acro-osteolysis is a rare presentation recognised by features of progressive distal phalanges resorption. The literature review aims to consolidate pre-existing understanding on the knowledge of the aetiology, pathophysiology, diagnosis, and management of acro-osteolysis.

Acro-osteolysis is distinguished by slow, progressive resorption of the distal phalanges in both the upper and lower limbs. Evidence to date demonstrates acro-osteolysis as a rare complication secondary to severe peripheral neuropathy in chronic diabetes.

Subtypes and Radiographic Features

The diagnostic gold standard for acro-osteolysis detection is through plain radiographs. Moreover, radiographic features help distinguish acro-osteolysis subtypes and associated causes. The first most common subtype is distal tuft resorption, which refers to erosion of the terminal phalangeal tufts. It is caused by chronic ischaemia, neuropathy, or inflammation. The second subtype is band acro-osteolysis (transverse acro-osteolysis) and involves erosion at the mid-shaft of the phalanges, often linked to chronic vascular compromise due to ischaemia and hypoxia. In longstanding or severe cases, a combined (confluent) pattern may occur, where both mid-shaft and distal tuft resorption are observed. This may represent disease progression from one form to another or a general worsening of the underlying pathology [1].

Acro-osteolysis is often detected incidentally through progressive clinical manifestations, including radiographic features such as terminal phalangeal resorption, soft tissue changes, and joint instability. Although it can present in asymptomatic patients, it can be identified based on clinical and radiographic features.

Aetiology and Differential Diagnoses

Case reports such as one by Limenis et al. (2021) link a few conditions that can possibly present with acro-osteolysis [1]. Genetic disorders include Hajdu-Cheney syndrome, Farber disease, Winchester syndrome, hereditary multicentric carpotarsal osteolysis, Joseph and Shinz acro-osteolysis, pycnodysostosis, and primary hypertrophic osteoarthropathy. Rheumatic disorders comprise psoriatic arthritis, rheumatoid arthritis, Raynaud’s disease, systemic lupus, sarcoidosis, and systemic sclerosis [2].

Other differentials that should be considered include hyperparathyroidism, neuropathies, local trauma (repeated strain injuries), thermal injury (frostbite, burns), and spinal dysraphism. It is rare that acro-osteolysis remains rare, which is characterised by rapidly extensive osteolysis with erosions [2-4].

To date, there is insufficient data to quantify the true prevalence of acro-osteolysis in diabetic patients, although an increasing number of cases show the pathology commonly presenting in the adult population. Its presentation can still span the age range, especially when there is an underlying rheumatologic pathology in children. Children, even though less likely to have developed acro-osteolysis due to environmental exposures, would be more likely to have underlying inherited genetic disorders [2].

Pathophysiology and Mechanisms

Causes and pathophysiology are still being explored. However, there are suggestions that active seropositive rheumatoid arthritis may directly contribute through the involvement of the RANK/RANKL system and pro-inflammatory cytokines [5]. One case report states that acro-osteolysis is associated with enhanced osteoclastogenesis and increased vascular endothelial growth factor [6]. Levels of osteoclast formation are associated with the severity of bone resorption.

Despite these diagnostic challenges, there is growing evidence linking acro-osteolysis to specific diabetic complications, including longstanding peripheral neuropathy (e.g., Charcot neuroarthropathy), chronic microvascular ischaemic changes, repeated unnoticed physical or thermal microtrauma, impaired autonomic and peripheral nervous systems, and heightened inflammatory cytokine activity, particularly tumour necrosis factor-alpha (TNF-α) and RANKL [2-4,7].

In low-oxygen tension, whether due to chronic ischaemia or inflammation, pathological bone resorption may be further accelerated. In diabetic vasculopathy, hypoxia promotes the formation and activation of osteoclasts derived from circulating monocytes. Local ischaemia is exacerbated by impaired perfusion and diminished vasodilation, both of which result from autonomic and sensory neuropathy. These processes are catalysed by RANKL and macrophage colony-stimulating factor (M-CSF). Hypoxia also enhances the expression of vascular endothelial growth factor (VEGF), which prolongs osteoclast survival and activity [6]. Chronic ischaemia can also trigger osteocyte apoptosis and disrupt normal bone remodelling, contributing to the pathogenesis of acro-osteolysis.

Diagnostic Challenges and Mimics

This under-recognition may be attributed to diagnostic challenges unique to diabetes. The condition’s radiographic and clinical features often overlap with more common diabetic foot complications. Additionally, the rarity and underreporting of acro-osteolysis, along with its broad differential diagnoses, including genetic, metabolic, rheumatologic, vascular, and environmental causes, make accurate diagnosis more difficult, especially when clinician awareness is low.

Radiographic findings such as terminal tuft resorption, soft tissue swelling, or osteolytic lesions may be misinterpreted as Charcot neuroarthropathy, chronic osteomyelitis, psoriatic arthritis, or changes secondary to diabetic sensory neuropathy. These are key mimics that can mask early pathological signs of acro-osteolysis, such as bone fragmentation, resorption, and deformity. Patients with diabetic peripheral neuropathy often experience diminished distal sensation, which may result in underreporting of early symptoms like pain, stiffness, or digital deformities.

Some experts also posit that distal acro-osteolysis in diabetic patients may represent an evolving or atypical manifestation within the broader spectrum of Charcot neuroarthropathy [8].

Clinical Impact and Outcomes

It is crucial to distinguish acro-osteolysis from more common clinical patterns of neuropathic osteoarthropathy (diabetic osteolysis), as seen in Charcot neuroarthropathy. Charcot neuropathy results from structural collapse and osteolysis of the mid- or hindfoot and causes joint subluxation, dislocations, and arch collapses. In contrast, acro-osteolysis is classed as an atypical atrophic form of diabetic osteopathy that affects the distal phalanges and leaves the distal joint spaces intact despite severe bone loss. This means patients retain a plantigrade foot without midfoot collapse and gross instability. However, diabetic acro-osteolysis is still serious due to its insidious pathogenesis.

Diabetic acro-osteolysis can cause more bone destruction than osteomyelitis. Overt infection and inflammation signs may be initially absent. This delayed progression can cause irreversible loss of bone architecture and altered weight-bearing mechanics, predisposing patients to ulcers and infection risks [9,10].

In a previous case of uncontrolled type 1 diabetes with severe neuropathy and recurrent fingertip ulceration, there was near-complete resorption of a distal phalanx and spontaneous auto-amputation. This illustrates that mutilating ulcerations in diabetic patients with acro-osteolysis can progress to open wounds and loss of phalanges if left unmanaged [11]. Once bone fragments and skin break down after resorption, secondary soft tissue infection can ensue, especially when there is altered load on a deformed toe.

Unlike Charcot arthropathy, diabetic acro-osteolytic distal phalanges remain structurally intact longer. Nevertheless, functional outcome remains guarded as these patients often have advanced neuropathy and are at high risk of wounds and amputations. Literature emphasises that patients experience an insidious course with minimal pain, until an ulcer or fracture draws attention [9-11].

Management Strategies

To prevent irreversible complications, physicians should maintain high suspicion when encountering diabetic patients with unexplained distal phalangeal bone loss on radiographs without classic infection signs. One study notes that diabetic acro-osteolysis can be distinguished from infection by the absence of systemic inflammation despite greater bone destruction. This recognition can help avoid unnecessary antibiotics or amputations [9].

Management is similar to Charcot protocols: conservative care through offloading the affected limb to reduce repetitive trauma [12]. This includes limiting weight-bearing with crutches or total contact casts. Other tactics involve podiatry care, patient education on daily foot inspection, and orthotic support such as custom insoles, extra-depth shoes, silicone toe caps, and protective padding.

A case report by Lin and Loh (2017) demonstrated the “Te technique” as a novel approach addressing both soft tissue and bone deformities in cases with soft tissue dissociation and bony non-union fracture instability [13].

Perfusion optimisation is essential in any diabetic foot presentation. Although acro-osteolysis can occur despite intact large-vessel circulation, microvascular flow is vital to regulate osteoclast activity. Peripheral vascular disease worsens outcomes by impairing tissue repair. Supportive measures include smoking cessation, lipid control, and possible use of vasodilators (e.g., cilostazol) [10]. Lumbar sympathectomy was historically attempted but is no longer recommended due to limited benefit [9].

Prognosis and Future Directions

The long-term prognosis of diabetic acro-osteolysis depends on prompt recognition and management. Reports posit diabetic osteolysis can be partially reversed or stabilised if the triggering causes are removed. Unlike infection (e.g., osteomyelitis), which requires antibiotics or surgery, neuropathic bone resorption can be halted if the limb is offloaded and protected. Early literature cautions against premature amputation in the absence of infection [9].

Although reversal has been documented in individual cases, this is not guaranteed. Many cases still progress to partial foot amputation, particularly after ulceration or musculoskeletal infection. This is seen in a report of a type 1 diabetic patient with poor control and multiple digits affected, where aggressive wound care still led to permanent shortening and deformity [2]. Preventable limb loss remains a key concern, as acro-osteolysis patients face an elevated risk of lower-limb amputation, similar to those with Charcot foot [11].

Current studies urge early recognition, careful follow-up, and management, as patients with acro-osteolysis, Charcot arthropathy, or neuropathic ulcers have significantly higher mortality, osteomyelitis, and amputation rates than those without such complications [14].

## Conclusions

This case illustrates a rare and progressive presentation of bilateral acro-osteolysis following osteomyelitis in a T2DM patient occurring in the context of chronic osteomyelitis. Despite multiple rounds of surgical intervention and appropriate antimicrobial therapy, imaging demonstrated ongoing bone resorption over more than a year. Vascular assessments showed no ongoing peripheral vascular disease. Extensive investigations have ruled out autoimmune and dermatological causes. Thus, these findings emphasise the importance of considering the diagnosis of acro-osteolysis in the context of persistent osteolytic processes and maintaining long-term radiological surveillance in complex diabetic foot infections.
